# S-Doped Sb_2_O_3_ Nanocrystal: an Efficient Visible-Light Catalyst for Organic Degradation

**DOI:** 10.1186/s11671-018-2522-5

**Published:** 2018-04-23

**Authors:** Hun Xue, Xinyi Lin, Qinghua Chen, Qingrong Qian, Suying Lin, Xiaoyan Zhang, Da-Peng Yang, Liren Xiao

**Affiliations:** 1 Fujian Key Laboratory of Pollution Control & Resource Reuse, Fujian Normal University, Fuzhou, 350007 China; 2 College of Chemical Engineering and Materials Sciences, Quanzhou Normal University, Quanzhou, 362000 China; 3 Fujian Provincial Key Laboratory of Ecology-toxicological Effects & Control for Emerging Contaminants, Putian University, Putian, 351100 China

**Keywords:** S-doped Sb_2_O_3_, Hydrothermal synthesis, Photocatalysis, Visible light, Organic degradation

## Abstract

The S-doped Sb_2_O_3_ nanocrystals were successfully synthesized using SbCl_3_ and thioacetamide (TAA) as precursors via a facile one-step hydrothermal method. The effects of pH of the precursor reaction solution on the product composition and property were determined. The results indicated that the doping amount of S could be tuned by adjusting the pH of the precursor solution. Furthermore, the S entered into the interstitial site of Sb_2_O_3_ crystals as S^2−^, which broadened the absorption wavelength range of the Sb_2_O_3_ nanocrystal. The S-doped Sb_2_O_3_ exhibited an excellent visible-light-driven photocatalytic activity in the decomposition of methyl orange and 4-phenylazophenol. Last, a possible photocatalytic mechanism of the S-doped Sb_2_O_3_ under visible light irradiation was proposed.

## Background

The semiconductor photocatalytic oxidation is an ideal environmental purification technique due to its utilization of solar energy, high stability, and nontoxicity. It can effectively remove organic pollutants, even at extremely low concentration, without causing any secondary pollution [1–6]. Among various kinds of semiconductor photocatalysts, TiO_2_ is widely investigated due to its excellent performance under UV irradiation on mineralization of a variety of organic compounds. However, the narrow band gap of TiO_2_ (3.2 eV) limits its utilization efficiency of solar energy [7, 8]. Therefore, developing the novel visible-light-driven photocatalysts is of great importance in environmental purification.

Currently, the visible-light-driven photocatalysts can be prepared via two major strategies: one is to develop new single-phase photocatalysts, such as CdS, Sn_2_Nb_2_O_7_, CaBi_2_O_4_, BiWO_4_, and SnIn_4_S_8_ [9–13], and the other one is to modify the UV-active photocatalysts. The modification can be realized by doping foreign elements, coupling UV-active photocatalysts with narrow band gap semiconductors, as well as forming inorganic-organic hybridization [14–30]. Sulfur (S), a non-metal element, is usually used to dope wide-band gap semiconductors, such as TiO_2_ [18–20], In(OH)_3_ [21], and Zn_2_SnO_4_ [22], to obtain the desired visible-light photocatalysts. However, the doping condition can significantly affect the valence state and form of S in the products, resulting in various photocatalytic activities. For example, Umebayashi et al. prepared the S-doped TiO_2_ by the oxidation annealing of TiS_2_, where the S atoms occupied the O atom sites in TiO_2_ to form Ti–S bonds [18]. Ohno et al. hydrolyzed titanium alkoxide in the presence of thiourea, and the hydrolysis product was calcined in the air to afford S^4+^ and S^6+^ substituted TiO_2_ [19]. Devi et al. prepared the S-doped TiO_2_ by a sol-gel method using sulfur powder as the S source. They found that S^6+^ was incorporated into the Ti^4+^ lattice of the TiO_2_ crystal [20]. S anion-doped Zn_2_SnO_4_ was prepared by calcining the mixture of thiourea and spinel Zn_2_SnO_4_ under argon atmosphere, during which S^2−^ ion entered into the interstitial site of Zn_2_SnO_4_ crystal [22].

The oxides and complex oxides of p-block metal antimony, such as Sb_2_O_3_ [31], M_2_Sb_2_O_7_ (M=Ca and Sr) [32, 33], NaSbO_3_ [32], Sr_1.36_Sb_2_O_6_ [34], ZnSb_2_O_6_ [35] and GaSbO_4_ [36], have unique crystal structures and electronic structures that can promote the photogenerated charge separation and reduce the recombination of photogenerated electrons and holes and thus have attracted considerable attentions as novel photocatalytic materials. However, most of them only respond to ultraviolet light, which limits their further applications. In the present work, S^2−^-doped Sb_2_O_3_ nanocrystal was prepared using SbCl_3_ and thioacetamide (TAA) as the sulfur source by a hydrothermal synthesis method. The effects of the S doping on the visible-light-driven photocatalytic activity of Sb_2_O_3_ for the degradation of methyl orange (MO) and 4-phenylazophenol were also evaluated. The results indicated that the S^2−^ doping was able to effectively narrow the band gap of Sb_2_O_3_ and thus improved the visible-light-driven photocatalytic activity of the Sb_2_O_3_. Our work provided a feasible synthesis route of the visible-light-responsive S-doped Sb composite oxide photocatalysts for efficient solar energy utilization.

## Methods

### Synthesis of S-Doped Sb_2_O_3_ Nanocrystals

All chemicals used in this work were purchased from Aladdin reagent and used directly. The S-doped Sb_2_O_3_ nanocrystals were synthesized by a hydrothermal method using SbCl_3_ and thioacetamide (TAA) as the precursors. Briefly, 3 mmol SbCl_3_ was added into a 100-mL Teflon-lined stainless steel autoclave reactor containing 70 mL deionized water and mechanically stirred for 15 min. Then, 4 mmol TAA was added into the mixed solution under constant stirring. The pH of the precursor solution was adjusted to 2, 5, 10, 12, and 14 using HCl or NaOH solution. The precursor solution was heated at 120 °C in an oven for 12 h. The produced precipitate was washed with distilled water and absolute ethanol several times and dried in the air at 70 °C. The products obtained at different pH were denoted as Sb_2_O_3_-S-pH (pH = 2, 5, 10, 12, and 14). The pure Sb_2_O_3_ was prepared in the absence of TAA using the similar procedure. Briefly, 3 mmol SbCl_3_ was dissolved in 50 mL deionized water and 20 mL absolute ethanol in a 100-mL Teflon-lined stainless steel autoclave reactor under vigorous stirring and heated at 120 °C for 12 h. The produced precipitate was washed with distilled water and absolute ethanol for several times. The obtained products were obtained after being dried at 70 °C.

### Material Characterization

The powder X-ray diffraction (XRD) patterns of the as-prepared Sb_2_O_3_ nanocrystals were recorded on a Bruker D8 Advance X-ray diffractometer using CuK_α_ radiation operated at the accelerating voltage of 40 kV and the applied current of 40 mA. The ultraviolet-visible diffuse reactance spectra (UV–vis DRS) were collected on a UV–vis spectrometer (Cary 500 Scan Spectrophotometers, Varian, USA) using BaSO_4_ as the reflectance standard. The transmission electron microscopy (TEM) and high resolution transmission electron microscopy (HRTEM) images were captured using a JEOL model JEM 2010 EX instrument operated at the accelerating voltage of 200 kV. X-ray photoelectron spectra (XPS) were recorded on a PHI Quantum 2000 XPS System equipped with a monochromatic Al K_α_ source and a charge neutralizer. The sample powder was ultrasonically dispersed in ethanol, and a drop of the suspension was dropped on a carbon film coated on a 3-mm-diameter fine-mesh copper grid. The C 1s peak at 284.8 eV of the surface adventitious carbon was used as the reference for all binding energies. Raman scattering spectra were obtained using a Renishaw inVia Raman microscope at room temperature.

### Photocatalytic Activity Measurements

The photocatalytic activity of the samples was carried out in a photoreaction vessel. A 500 W halogen lamp (Philips Electronics) was positioned beside the cylindrical reaction vessel with a plane side as the visible light source. Two cut-off filters of 420 and 800 nm were placed between the lamp and the vessel to ensure only visible light passed to reach the vessel. The vessel was maintained at room temperature by circulating water. The photocatalyst (80 mg) was powdered and added to the vessel containing 80 mL 3 × 10^−5^ mol L^−1^ MO or *p*-hydroxyazobenzene (1.2 × 10^−4^ mol L^−1^) aqueous solution. The mixture was stirred in the dark for 1 h to reach the adsorption/desorption equilibrium on the photocatalyst and then exposed to the visible light. A 4 mL suspension was taken at certain time intervals and centrifuged. The supernatant was collected and measured with a Shimadzu UV-1750 UV–Vis–NIR spectrophotometer. The absorbance at the maximum absorption was recorded.

## Results and Discussion

**Fig. 1:**
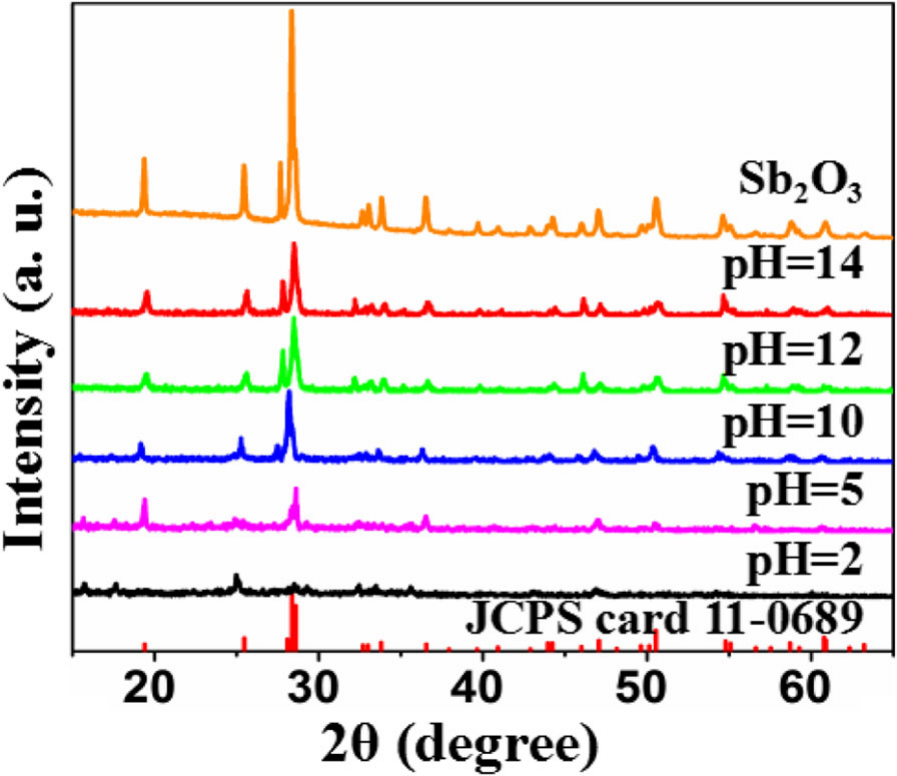
XRD patterns of Sb_2_O_3_ and S-doped Sb_2_O_3_ synthesized at various pHs (pH = 2, 5, 10, 12, and 14)

The XRD patterns of the control Sb_2_O_3_ and Sb_2_O_3_-S-pH (pH = 2, 5, 10, 12, and 14) are shown in Fig. 1. All peaks of both control sample and Sb_2_O_3_-S-pH (pH = 10, 12, and 14) were indexed to Sb_2_O_3_ (JCPDS card 11-0689). The low pHs (2 and 5) of the precursor solution lowered the crystallinity of the product and caused the formation of impure phases. These observations indicated that the pH of precursor solution could significantly affect the composition of the products, and the pure Sb_2_O_3_ phase could only be obtained under alkaline conditions. The intensities of all characteristic XRD peaks of Sb_2_O_3_ were higher than those of Sb_2_O_3_-S-pH (pH = 10, 12, and 14), indicating that TAA inhibited the growth of Sb_2_O_3_ crystallite.

**Fig. 2:**
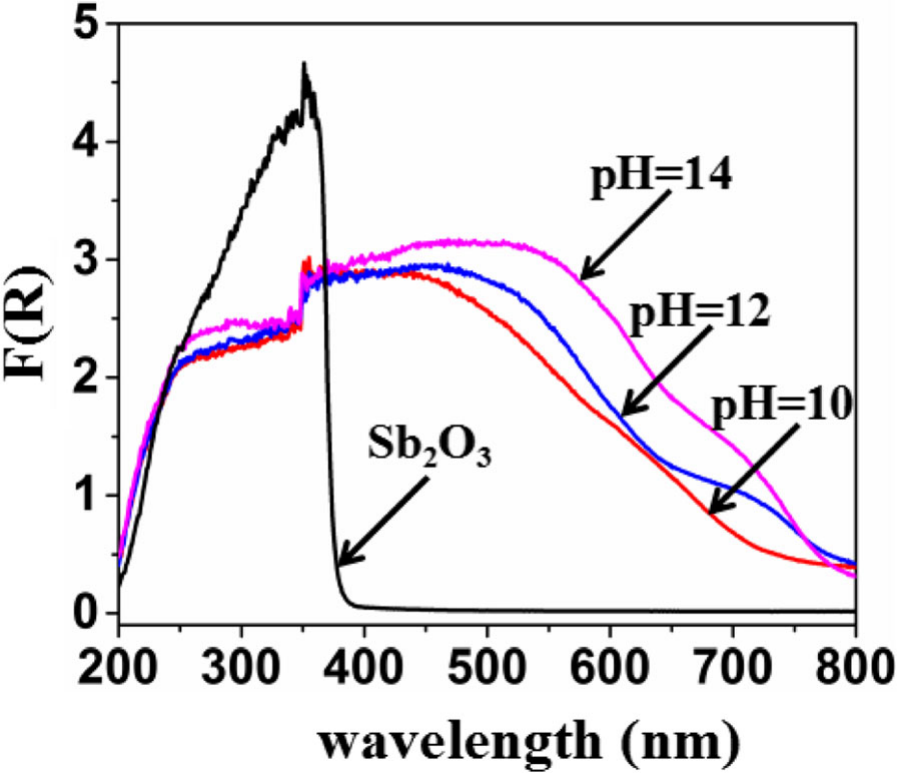
Diffuse reflectance absorption spectra of Sb_2_O_3_ and Sb_2_O_3_-S-pH (pH = 10, 12, and 14)

Figure 2 shows the UV–vis diffuse reflectance spectra of Sb_2_O_3_ and Sb_2_O_3_-S-pH (pH = 10, 12, and 14). The maximum absorption of Sb_2_O_3_ appeared at ca. 380 nm, suggesting that Sb_2_O_3_ only responded to UV irradiation. Compared with that of pure Sb_2_O_3_, the band-gap transitions of Sb_2_O_3_-S-pH (pH = 10, 12, and 14) exhibited obvious redshifts, and the redshift increased with the increase of the pH of the precursor solution. Therefore, it is reasonable to believe that the band gap narrowing was dominantly attributed to the S doping. The S 3p states mixed with valence band (VB), which increased the width of VB and lowered the energy shift in the optical absorption [18]. It is well known that TAA can be hydrolyzed to generate S^2−^ in an alkaline solution and the increased pH can promote the production of S^2−^. Therefore, the increases in the redshift of the band-gap transition with the pH of precursor solution might be attributed to the increased amounts of S doped in Sb_2_O_3_ at higher pHs. Therefore, the S doping amount can be controlled by simply adjusting the pH of the precursor solution.

**Fig. 3:**
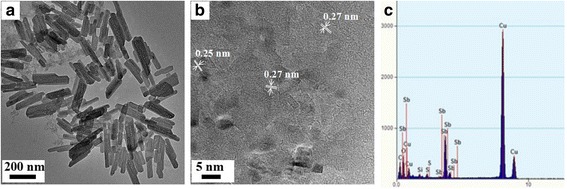
**a** TEM. **b** HRTEM images and **c** EDS spectrum of Sb_2_O_3_-S-12

TEM was done to identify the morphology and crystal structure of S-doped Sb_2_O_3_. As shown in Fig. 3a, the Sb_2_O_3_-S-12 was rod-shaped with a diameter of ~ 40 nm and length ranging from 100 to 200 nm. The HRTEM image revealed that the nanorods were consisted of many randomly assembled nanoparticles with an average diameter of ~ 5 nm (Fig. 3b). Clear diffraction patterns with interplanar distances of 0.25 and 0.27 nm were observed in the HRTEM images, which can be assigned to the (200) and (131) planes of Sb_2_O_3_, respectively. The EDS analysis (Fig. 3c) revealed that Sb, O, and S elements existed in the samples, indicating that the S-doped Sb_2_O_3_ was successfully prepared.

**Fig. 4:**
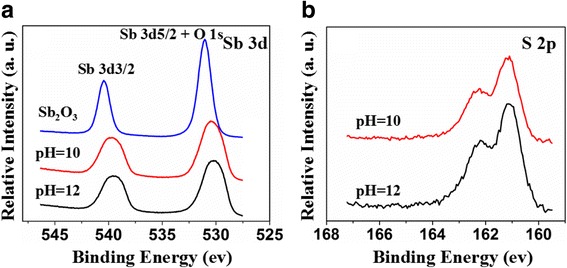
XPS spectra of Sb_2_O_3_ and Sb_2_O_3_-S-pH (pH = 10 and 12). **a** Sb 3d. **b** S 2p

The XPS spectra and high-resolution Sb 3d and the O 1s XPS spectra of Sb_2_O_3_ and Sb_2_O_3_-S-pH (pH = 10 and 12) are displayed in Fig. 4a. The Sb 3d 3/2 peak of Sb_2_O_3_ appeared at 539.8 eV and the peak at 530.5 eV were assigned to Sb 3d 5/2 and O 1s, suggesting that the oxidation state of Sb is + 3 instead of + 5 with slightly higher binding energies [37, 38]. The S doping reduced the binding energies of Sb 3d, yet showed no significant effects on the chemical state of Sb. These results indicated that the S doping changed the chemical environments of the Sb ions and increased the electron densities around the Sb ions due to the lower electronegativity of S [39]. Compared with Sb_2_O_3_-S-10, Sb_2_O_3_-S-12 contained more S. The electron density around its Sb was higher than that of Sb_2_O_3_-S-10, and thus, the Sb 3d binding energy of Sb_2_O_3_-S-12 shifted towards low energy direction. The S 2p high-resolution XPS spectra in Fig. 4b revealed two peaks at 161.5 and 162.7 eV which were attributed to S^2−^ [22, 40, 41]. The radius of S^2−^ (184 pm) is much greater than that of O^2−^ (132 pm). Therefore, it was difficult for S^2−^ to replace the O^2−^ in Sb_2_O_3_ [22, 42]. It is most likely that S^2−^ entered into the interstitial site of the Sb_2_O_3_ crystal [18]. XPS analysis indicated that Sb_2_O_3_-S-12 contained more S than Sb_2_O_3_-S-10, further confirming that the pH of the precursor solution could be used to control the S doping amount.

**Fig. 5:**
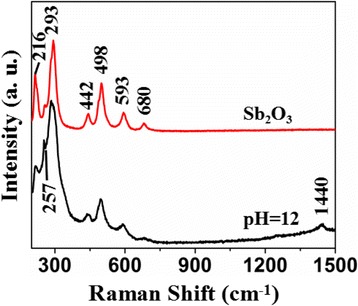
Raman spectra of Sb_2_O_3_ and Sb_2_O_3_-S-12

Figure 5 shows the Raman spectra of the Sb_2_O_3_ and Sb_2_O_3_-S-12. Sb_2_O_3_ exhibited signal at 216, 257, 293, 442, 498, 593, and 680 cm^−1^. A new peak appeared at 1440 cm^−1^ in the spectra of Sb_2_O_3_-S-12, which might be the result from the S doping. In addition, compared to the peaks of Sb_2_O_3_, the peak width of the peaks of Sb_2_O_3_-S-12 increased and the symmetry of their peak shapes decreased, indicating that the S doping increased the internal defects [43]. The S doped into the interstitial site of Sb_2_O_3_ crystals caused the deformation of the lattice.

**Fig. 6:**
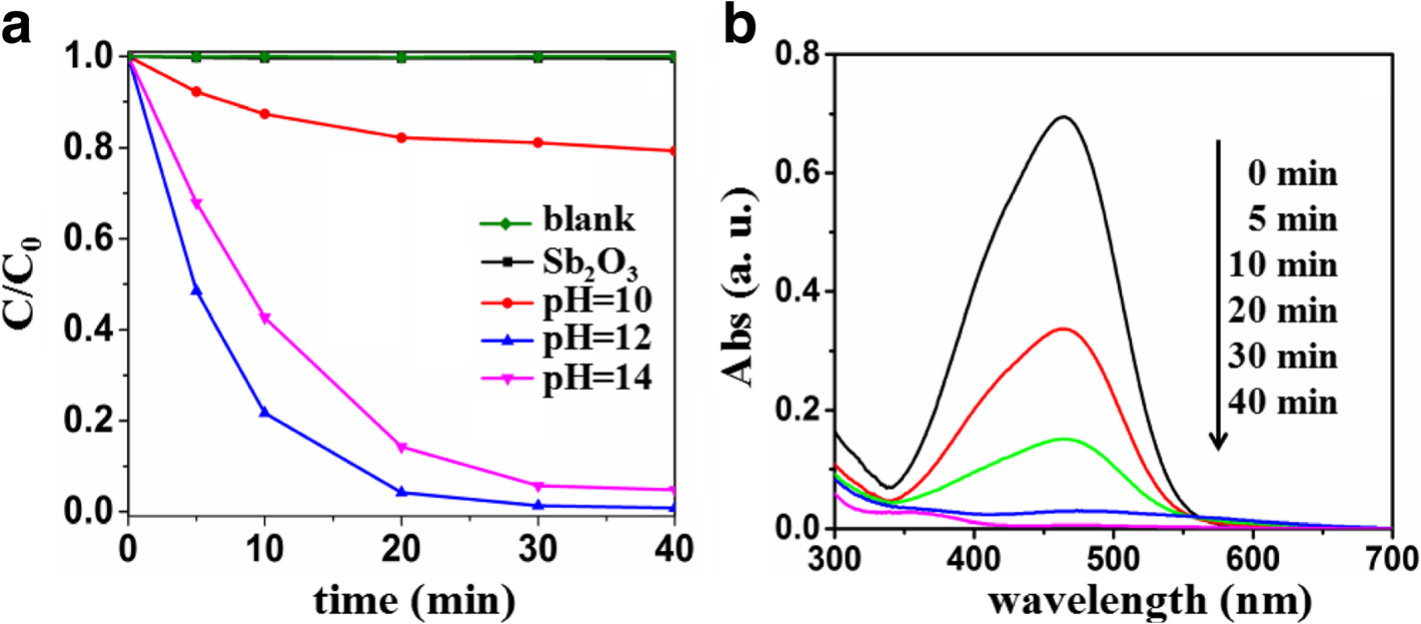
**a** Temporal changes of MO concentration as monitored by the UV–vis absorption spectra at 464 nm on Sb_2_O_3_ and Sb_2_O_3_-S-pH (pH = 10, 12, and 14). **b** Temporal absorption spectral patterns of MO during the photodegradation process over Sb_2_O_3_-S-12

The visible-light-driven photocatalytic activities of pure and S-doped Sb_2_O_3_ for the degradation of MO were determined. The temporal changes in the MO concentration were monitored by measuring the UV–vis absorption of the MO solution over the photocatalyst at 464 nm (Fig. 6a). MO appears very stable under visible light with almost no degradation. Since the Sb_2_O_3_ could not be excited under visible light, the MO degradation was negligible in the presence of Sb_2_O_3_. Doping with certain amount of S would enhance the visible light absorption, increase the internal defects, and promote the separation rate of the photogenerated electron-hole pairs. As a result, the visible-light-driven photocatalytic activity of the samples could be improved, which can be demonstrated by the S-doped Sb_2_O_3_. It is noteworthy that the photocatalytic activities of the S-doped Sb_2_O_3_ prepared at different pHs were different. Sb_2_O_3_-S-10 exhibited the lowest visible-light-driven photocatalytic activity. It can be explained that the hydrolysis of TAA under weak alkaline conditions is weak, producing less S^2−^. The Sb_2_O_3_-S-14 with more S produced under strong alkaline condition exhibit a lower photocatalytic activity compared with the Sb_2_O_3_-S-12 with relatively less S, which may be attributed to the excessive S doping causing too many defects that were the recombination centers of the photogenerated electron-hole pair. Compared with the counterparts, Sb_2_O_3_-S-12 exhibited the highest visible-light-driven photocatalytic activity and was able to degrade 99.2% MO in 40 min under visible light irradiation. Figure 6b shows the temporal absorption spectral patterns of MO during the photodegradation over Sb_2_O_3_-S-12. The absorption of MO was at 464 nm, which was attributed to its –N=N– unit. The absorption intensity decreased as the visible light irradiation proceeded, indicating that the –N=N– double bonds were gradually decomposed [44].

**Fig. 7:**
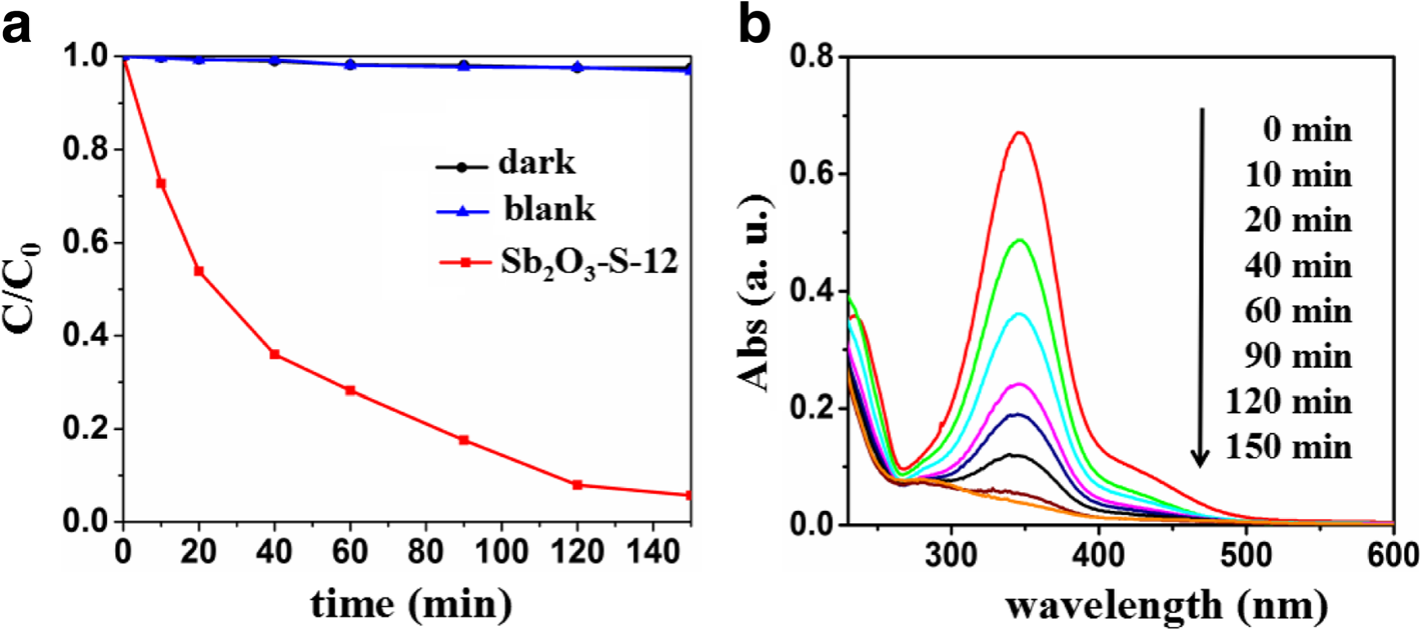
**a** Temporal changes of 4-phenylazophenol concentration as monitored by the UV–vis absorption spectra at 347 nm on Sb_2_O_3_-S-12. **b** Temporal absorption spectral patterns of 4-phenylazophenol during the photodegradation process over Sb_2_O_3_-S-12

The photocatalytic performance of Sb_2_O_3_-S-12 for the degradation of p-hydroxyazobenzene was also determined. Figure 7a, b presents the photocatalytic activity of Sb_2_O_3_-S-12 for the p-hydroxybenzobenzene degradation and the corresponding UV–vis absorption spectra of p-hydroxybenzobenzene during the degradation. No p-hydroxyazobenzene degradation was observed under the visible light irradiation in the absence of Sb_2_O_3_-S-12. In contrast, 94.3% of p-hydroxyazobenzene was degraded under the visible light irradiation for 150 min in the presence of Sb_2_O_3_-S-12. In addition, the absorption of p-hydroxybenzobenzene at 347 nm decreased as the irradiation prolonged. These results indicated that S-doped Sb_2_O_3_ could be implemented in the degradations of different pollutants.

**Fig. 8:**
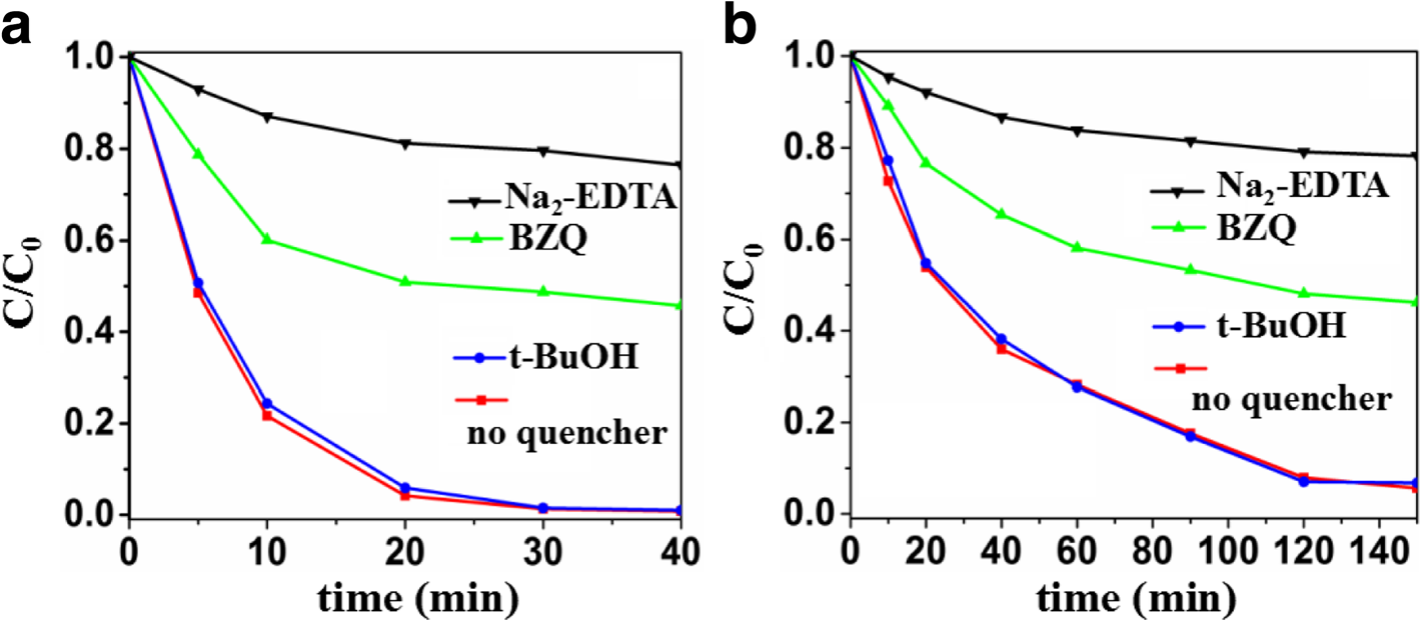
Trapping experiment of active species over Sb_2_O_3_-S-12 during the photocatalytic degradation of **a** MO. **b** 4-Phenylazophenol

To explore the photocatalytic mechanism of the S-doped Sb_2_O_3_ nanocrystals, the major oxidative species in the photocatalytic reaction were trapped using *p*-benzoquinone (BZQ, an O_2_^−^· radical scavenger), disodium ethylene diamine tetra acetate (Na_2_-EDTA, a hole scavenger), and tert-butanol (t-BuOH, a OH· radical scavenger) [39, 45]. As shown in Fig. 8, the addition of t-BuOH showed no deleterious effect on the photocatalytic activity of Sb_2_O_3_-S-12 and the presence of BZQ or Na_2_-EDTA decelerated significantly its photocatalytic degradation efficiency of MO and 4-phenylazophenol. Therefore, it can be concluded that h^+^ and O_2_^−^· radicals were the dominant oxidative species of the S-doped Sb_2_O_3_ photocatalysis and the OH· radical was not involved.

**Scheme 1:**
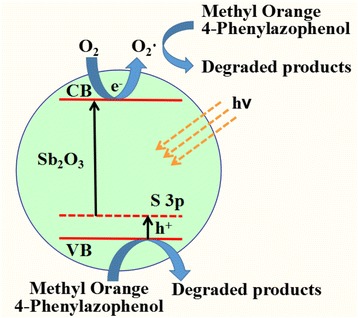
Possible mechanism of the photocatalytic degradation of MO or 4-phenylazophenol over Sb_2_O_3_-S-12 visible-light photocatalyst

Based on these discussions, a possible mechanism for photocatalytic degradation of MO over S-doped Sb_2_O_3_ nanocrystals was proposed as follows (Scheme 1). S-doped Sb_2_O_3_ can be efficiently excited to create holes in VB and electrons in conduction band (CB) under visible light irradiation. The S doping increased the VB width of Sb_2_O_3_. The electrons are long-lived enough to react with adsorbed O_2_ to produce O_2_^−^· radicals. The photogenerated h^+^ and O_2_^−^· exhibited a strong oxidation potential which can directly oxidize MO and 4-phenylazophenol to degradated products.

## Conclusions

The S-doped Sb_2_O_3_ nanocrystals were successfully prepared from SbCl_3_ and TAA via a facile one-step hydrothermal method under alkaline conditions. S entered into the interstitial site of Sb_2_O_3_ crystals as S^2−^, which broadened its visible light absorption range. The pH of the precursor solution can significantly affect the S doping amount, which further alternates the visible-light-driven photocatalytic activity of the S-doped Sb_2_O_3_ nanocrystal. The nanocrystal prepared at pH 12 exhibited the highest visible-light-driven photocatalytic activity and was able to degrade 99.2% MO and 94.3% p-hydroxybenzobenzene in 40 and 150 min, respectively, under visible light irradiation. The visible-light photocatalytic degradation of MO and *p*-hydroxyazobenzene by S-doped Sb_2_O_3_ were achieved by h^+^ and O_2_^−^·.

## Abbreviations

BZQ: *p*-Benzoquinone
MO: Methyl orange
Na_2_-EDTA: Disodium ethylene diamine tetra acetate
S: Sulfur
TAA: Thioacetamide
t-BuOH: Tert-butanol
